# A survey of antimicrobial resistance in Enterobacteriaceae isolated from the Chesapeake Bay and adjacent upper tributaries

**DOI:** 10.1002/mbo3.839

**Published:** 2019-04-04

**Authors:** Stefan Riedel, Nicholas Boire, Kathryn A. Carson, Aravinda Vadlamudi, Joshua Khuvis, Vivek Vadlamudi, Vajini Atukorale, Victoria A. A. Riedel, Nicole M. Parrish

**Affiliations:** ^1^ Department of Pathology Beth Israel Deaconess Medical Center Boston Massachusetts; ^2^ Harvard Medical School Boston Massachusetts; ^3^ Division of Microbiology, Department of Pathology, School of Medicine The Johns Hopkins University Baltimore Maryland; ^4^ Department of Epidemiology Johns Hopkins Bloomberg School of Public Health Baltimore Maryland

**Keywords:** antibiotic pollution, antimicrobial resistance, Chesapeake Bay, Enterobacteriaceae, One Health

## Abstract

In recent years, the rise in antimicrobial resistance (AR) in the healthcare setting as well as the environment has been recognized as a growing public health problem. The Chesapeake Bay (CB) and its upper tributaries (UT) is a large and biologically diverse estuary. This pilot study evaluated the presence of AR of gram‐negative bacteria isolated from water samples collected at various sites of the Chesapeake Bay. Bacterial organisms were identified and antimicrobial susceptibility testing was performed by phenotypic and genotypic methods. Ninety‐two distinctly different gram‐negative bacteria were identified; *Klebsiella pneumoniae*, *Enterobacter cloacae*, *Enterobacter aerogenes*, *Serratia marcescens*, and *Escherichia coli* were most often isolated. *Serratia marcescens *was more frequently isolated in samples from the UT compared to the CB. Antimicrobial resistance was more frequently detected in organisms from the CB by phenotypic and genotypic methods. Antimicrobial resistance to ampicillin, imipenem, tetracycline, and chloramphenicol were the most frequently observed resistance patterns. ACT‐1, CMY, and SHV genes were the most frequently detected resistance genes, with predominance in organism isolated from the CB. The results from this study emphasize the importance for further developing comprehensive surveillance programs of AR in bacterial isolates in the various environments, such as recreational and other water systems.

## 1 INTRODUCTION

During the recent two decades, antimicrobial resistance (AR) in bacteria has been recognized as a critical public health problem (Hawken & Snitkin, 2019). Besides the fundamental utility of antibiotics in improving human health, antibiotics are widely used for treatment and prevention of infections in animals and plants, as well as for promoting growth in animal farming (Cabello, 2006; McManus, Stockwell, Sundin, & Jones, 2002; Singer et al., 2003; Smith, Harris, Johnson, Silbergeld, & Morris, 2002). However, during the past two decades, development and spread of AR in many bacteria has been recognized with increasing frequency, and now presents a global health crisis (Hawken & Snitkin, 2019; Wattkins & Bonomo, 2016). Each year in the United States alone, approximately 2 million infections due to AR bacteria occur, resulting in at least 23,000 deaths (Centers for Disease Control & Prevention, 2013). The economic impact of AR is tremendous and healthcare costs due to infections with AR bacteria continue to increase and pose a significant burden on societies (Wattkins & Bonomo, 2016). While AR has been described in almost all bacterial pathogens, AR and specifically emerging multidrug resistance (MDR) among gram‐negative bacteria represents a unique and immediate threat (Hawken & Snitkin, 2019; Lautenbach & Perencevich, 2014). Furthermore, gram‐negative bacteria and their associated AR have certainly the highest implication for human health, and would be expected to be most likely acquired from external sources. The development of AR is a complex process that is driven by multiple factors, including overuse of antimicrobial agents in healthcare, inadequate adherence to infection control practices, global travel and tourism, antimicrobial overuse in agriculture, and poor sanitation and contaminated water systems (Wattkins & Bonomo, 2016). In recent years, these complex factors contributing to AR have been well recognized and become important components of monitoring AR evolution in the greater context of global health, as exemplified in One Health Initiative (2018).

Previous studies and reports suggested that as much as 80% of the total production of antimicrobial agents in the United States is used in agriculture, animal farming, and veterinary medicine (Aarestrup, 1999; Bates, Jordens, & Griffiths, 1994; Ferber, 2003; FDA, 2014; NRC, 1999; Witte, 1998). Furthermore, a significant portion of those antimicrobial agents used in agriculture and animal husbandry are also important antimicrobial agents used for the treatment of common infections in humans (FDA, 2014; vanBoeckel et al., 2015). Therefore, it comes as no surprise, that the appearance of AR is directly linked to the use and overuse of antimicrobial agents; this fact was previously described for clinical healthcare settings as well as veterinary medicine and farming (Aarestrup, 1999; Bates et al., 1994; Chantziares, Boyen, Callens, & Dewulf, 2014; Ferber, 2003; NRC, 1999; Singer et al., 2003; Smith et al., 2013; vanBoeckel et al., 2015; Witte, 1998). In addition, the World Health Organization (WHO) previously recognized that the use of antimicrobial agents in animals clearly affects the occurrence of AR in bacteria responsible for infections in humans (Martinez, 2009; WHO, 2000). While it is important to acknowledge that many of the antimicrobial agents currently used in human healthcare settings were initially discovered as compounds produced by various environmental microorganisms, it is equally important to understand that many of the AR genes in human pathogenic microorganisms, commonly acquired by horizontal gene transfer, also originated in environmental organisms (Aminov, 2009; Aminov & Mackie, 2007; Campagnolo et al., 2002; Martinez, 2009). Therefore, it seems intuitive that environmental pollution by antimicrobial agents and their residues serve as a contributing factor in the evolution of AR in natural microbial ecosystems (Martinez, 2009). In addition, the run‐off from agriculture, hospitals and other healthcare settings, as well as wastewater treatment plants, among other sources, may also contribute to increased presence of antimicrobial agents, their residues, as well as pathogenic bacteria (Aminov, 2009; Aminov & Mackie, 2007; Börjesson, Matussek, Melin, Löfgren, & Lindgren, 2010; Campagnolo et al., 2002; Hooda, Edwards, Anderson, & Miller, 2000; Kulkarni et al., 2017; Kümmerer, 2009; Martinez, 2009; Rosenberg Goldstein et al., 2012). However, despite the growing number of studies and associated evidence, little is known about the overall effects of antimicrobial agents on the dynamics of the various larger ecosystems or the microspheres within such systems.

We previously described unusual resistance patterns in bacteria implicated in wound infections in patients seen within our hospital network (Parrish, Luethke, Dionne, Carroll, & Riedel, 2011). The infections were related to recreational activities on the Chesapeake Bay waters. These findings and others alike reported in the literature (Ceccarelli et al., 2015; McNicol et al., 1980; Morgan, Guerry, & Colwell., 1976; Shaw et al., 2014; Shaw, Sapkota, Jacobs, He, & Crump, 2015), were the basis to conduct a pilot survey of the Chesapeake Bay and upper tributaries to assess bacterial diversity and AR. Specifically, this study evaluated antimicrobial susceptibility patterns for enteric gram‐negative bacteria isolated from water samples obtained from various locations of the Chesapeake Bay and its upper tributaries in Maryland. Our findings provide a more comprehensive analysis of such a kind for bacterial AR in gram‐negative bacteria in the Chesapeake Bay area.

## 2 MATERIALS AND METHODS

**Figure 1:**
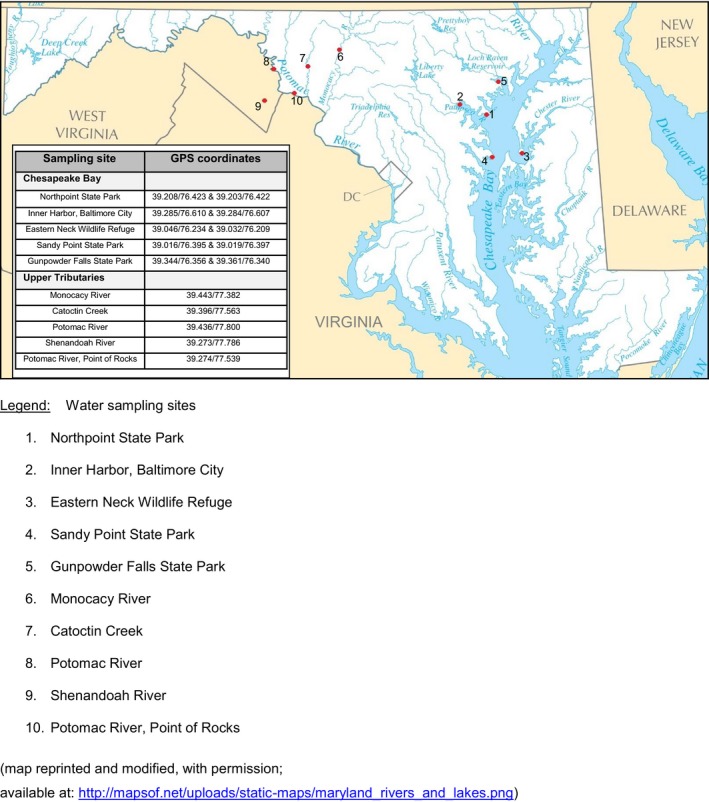
Water sampling sites and global positioning system 1 (GPS) coordinates for surface water samples collected from the Chesapeake Bay and adjacent Upper Tributaries

We conducted a pilot survey of the Chesapeake Bay and its upper tributaries in July 2012 to assess the presence of AR in enteric gram‐negative bacteria. Considering the rapid emergence of AR in gram‐negative bacteria and the associated increasing risk to human health, as described above, we focused this pilot survey initially on isolation of gram‐negative bacteria. Water samples were collected at 10 locations as outlined in Figure 1. These 10 sites were selected based on those included in a previous survey conducted and published in 1976 (Morgan et al., 1976), therefore serving as a comparative reference; in addition, sites were selected because of their close proximity to industrial agriculture, human habitation, and wastewater treatment plants. Specifically, Sandy Point State Park, Northpoint State Park, and Gunpowder Falls State Park were chosen as sites with frequent human recreational use. The Sandy Point State Park is located on the western shore of the mid‐bay region and at the base of the Chesapeake Bay Bridge; it also includes an artificial beach. The park is open to the public year‐round with a high volume of visitors on an annual basis. Northpoint State Park is located immediately north of the mouth of the Patapsco River, which serves as the entrance to the Baltimore Inner Harbor. The park features an unguarded waterfront that is open to waders and swimmers, in addition to biking and hiking trails, as well as two fishing piers. The Gunpowder Falls State Park is located at the western coast of the northern part of the Chesapeake Bay in Baltimore and Harford counties. The park has varied topographies, ranging from tidal wetlands, rugged and steep shores, as well as a natural swimming beach. The park is frequent to human recreational activities, including swimming, kayaking, canoeing, and fishing. The Eastern Neck Wildlife Refuge is an island located at the eastern shore of the mid‐region of the bay; it is a habitat for thousands of wintering waterfowl. The refuge is characterized by a variety of habitats, including brackish marsh, natural ponds, grasslands, and upland forests. Samples were collected at the immediate waterfront of the bay. The Baltimore Inner Harbor is located at the mouth of the Jones Falls, creating the wide and short northwest branch of the Patapsco River. Parts of the area along the harbor have been developed with condominiums, retail space, restaurants, and hotels. The harbor serves as a major tourist attraction, including historic ships at anchor, piers for cruise ships, water taxis, and other tourist water activities. All of the collection sites in the upper tributaries are located in the State of Maryland, with the exception of the site at the Shenandoah River, which is in West Virginia. All sites in the upper tributaries were in close proximity to agricultural and farming operations. For each of these ten locations, global positioning system (GPS) coordinates were recorded for each sampling site as shown in Figure 1. Water sampling, sample processing, organism identification, and antimicrobial resistance testing were performed as described in the following detailed procedures. One sampling site located at the Point of Rocks, on the Potomac River, was located immediately downstream from the Point of Rocks wastewater treatment facility, whereas the other Potomac River site, was located immediately upstream from the Shepherdstown wastewater treatment facility.

### 2.1 Water sampling procedure

For the collection sites in the Chesapeake Bay, surface water samples were collected just below the surface using 500‐ml sterile, glass, screw‐capped bottles that were opened immediately after being submerged in the water. Sampling was performed in two steps; one sample was collected within a few meters from the shore (proximal sample), and a second sample was collected further away from the shore, at a distance of approximately 150 m (distal sample). Sampling at the upper tributary sites (i.e., rivers and creeks) was performed in a similar fashion; however, only one sample was collected several meters into the river, where the water was deeper and the current was more rapid than in closest proximity to the riverbank. All personnel collecting water samples wore clean gloves to avoid contamination of bottles and/or samples with human skin flora. Upon completion of the collection process at each site, all samples were placed on ice and transported back to our laboratory for further processing.

### 2.2 Physical and chemical water quality measurements

Water column depth, water temperature, and water pH were measured on every sampling date and at every sampling collection.

### 2.3 Water and sample processing

Water samples received at the laboratory were poured through glass filtration units, using vacuum filtration through presterilized 0.2‐µm bacterial recovery filters (Millipore, Billerica, MA). Subsequently, each membrane filter was cut into sections using sterile scissors and forceps; the sections were then placed into Trypticase soy broth (Becton, Dickinson & Co., Sparks, MD) and incubated at 35°C for 24 hr. After the initial incubation period, aliquots from broth tubes exhibiting turbidity were subcultured onto sheep blood agar and MacConkey agar. Agar plates were incubated at 35°C in 5% CO_2_ for 24 hr. Broth tubes that did not demonstrate turbidity at 24 hr were re‐incubated for an additional 24‐hr period. If these broth tubes did not demonstrate turbidity after the additional incubation period, subcultures onto sheep blood agar were performed before the sample was deemed negative for bacterial growth.

### 2.4 Organism identification and antimicrobial resistance testing

All distinct colonies recovered from positive broth cultures and on the initial agar media were further processed for organism identification. Organism identification was performed using routine microbiological methods, including use of additional differential and selective agar media (e.g., Hektoen enteric agar, Columbia agar with colistin and nalidixic acid, and MacConkey agar), Gram stain, various bench tests (e.g., catalase, oxidase), and commercial bench identification methods, including the API 20E and API 20NE tests (bioMérieux). All tests were performed according to standard microbiology laboratory procedures and manufacturers instruction manuals. Final confirmation for identification of all bacterial isolates to the species level was done using matrix‐assisted‐laser‐desorption ionization time‐of‐flight mass spectroscopy (MALDI‐TOF MS; Bruker Daltonics, Billerica, MA), an FDA cleared platform for the identification of gram‐negative and gram‐positive bacteria, as well as anaerobic bacteria and yeasts of clinical significance. For all isolates, a log confidence score of ≥2.0 was required for identification to the species level.

Antimicrobial susceptibility testing (AST) was performed for all gram‐negative enteric bacteria as well as gram‐negative nonfermentative bacteria, using disk‐diffusion and E‐test methods, following CLSI guidelines and standard laboratory procedures (CLSI, 2012,2016), determining either zone diameters (mm) for growth inhibition or minimum inhibitory concentrations, MICs, (µg/ml). These results were then interpreted as susceptible [S], intermediate‐susceptible [I], or resistant [R], using the CLSI M100‐S25 guidelines and performance standards for AST (CLSI, 2016). The “resistant” category implies that bacterial isolates are not inhibited by the usually achievable concentrations of an antimicrobial agent. The “susceptible” category implies that bacterial isolates are inhibited by the usually achievable concentration of an antimicrobial agent. The “intermediate” category includes isolates with MICs that approach the usually achievable drug concentration in blood for a specific bacterial isolate; this category therefore implies that the clinical efficacy of the particular antimicrobial agent may be diminished based on its pharmacokinetic properties and the specific site of an infection. For clinical purposes, when treating patients with infections and considering AST results, it is common practice to select antimicrobial agents that tested “S” for treatment; however, antimicrobial agents that resulted in the interpretations “R” are not considered for treatment. It is furthermore common practice to consider antimicrobial agents that tested “I” as being less suitable for treatment, unless special clinical circumstances (e.g., multidrug‐resistant bacteria and difficult to treat infections) may warrant the use of such antimicrobial agents. From a clinical perspective and for surveillance studies, the AST categories “I” and “R” are frequently combined because neither category would be commonly considered appropriate for primary clinical use for treatment of infections. The following antimicrobial agents were chosen for AST: ampicillin; ceftriaxone; ceftaroline; cefepime; piperacillin/tazobactam; imipenem; meropenem; ertapenem; doripenem; ciprofloxacin; gentamicin; tobramycin; chloramphenicol; and tetracycline; trimethoprim/sulfamethoxazole. These antimicrobial agents represent those commonly used in clinical patient care for the treatment of infections due to gram‐negative bacteria; representative agents from each antimicrobial agent class were chosen, based on availability for the chosen testing method as well as availability for interpretive guidelines.

In addition, detection of common AR genes was performed by OpGen Clinical Services Laboratory using the Acuitas Resistome^®^ Test (OpGen Inc., Gaithersburg, MD). This test is a microfluidic PCR array that analyzes culture isolates from gram‐negative bacilli for approximately 50 antibiotic resistance gene families across several hundred variants associated with multidrug‐resistant organisms (MDROs) including genes for carbapenemases, extended‐spectrum β‐lactamases (ESBL), and *amp*C β‐lactamases. The Acuitas Resistome^®^ Test specifically tests for the following resistance genes: ACT‐1/MIR‐1, CMY‐2/CFE‐1, and CMY‐70/CFE‐1, which are plasmid‐mediated *AmpC* β‐lactamases (cephamycinases), belonging to the Bush‐Jacoby group 1 of β‐lactamases (Bush & Jacoby, 2010). ACT‐1/MIR‐1 is typically carried by *Escherichia coli* and *Klebsiella pneumoniae* in the US, and is homologous with chromosomal *AmpC*‐genes in *Enterobacter cloacae*. CMY‐2/CFE‐1 is a large family of plasmid‐mediated *AmpC* β‐lactamases typically carried by *Escherichia coli* and *K. pneumoniae* frequently isolated in the Europe and Asia; CMY‐70/CFE‐1 are plasmid‐mediated *AmpC* β‐lactamases found in *Citrobacter freundii* and *K. pneumoniae*. CMY‐47 encodes for a large family of chromosomal‐encoded *AmpC* β‐lactamases; DHA‐1 encodes for a large family of plasmid‐mediated *AmpC* β‐lactamases, frequently isolated in *Salmonella enteritidis* and *Morganella morganii*. Mox‐1/CMY‐1 encodes for large families of homologous plasmid‐mediated *AmpC* β‐lactamases; OXA‐50 is a chromosomal‐encoded oxacillinase, identified in clinical strains of *Pseudomonas aeruginosa*. SHV‐G238/E240 and SHV‐G156 encode for large families of chromosomal‐ as well as plasmid‐mediated β‐lactamases that hydrolyze narrow‐spectrum cephalosporins, penicillins, and aztreonam; these β‐lactamases can be inhibited by clavulanic acid (Barnaud et al., 1998; Bush & Jacoby, 2010; Chaves et al., 2001; Ghafourian, Sadeghifard, Soheili, & Sekawi, 2015; Girlich, Naas, & Nordmann, 2004; Philippon, Arlet, & Jacoby, 2002; Queenan & Bush, 2007). In brief, 0.5 McFarland standards were prepared from colony isolates grown overnight on a sheep blood agar plate. Nucleic acid extraction was done from 500 µl of each McFarland standard using the Roche MagNA Pure 96 DNA and Viral NA Large Volume Kit (P/N 06374891001) on the MagNA Pure 96 System. PCR was performed using primers and fluorescent reporter probes (Applied Biosystems Custom TaqMan^®^ MGB™ Probes with 5’‐FAM™ and a 3’ non‐fluorescent quencher). All PCRs used dUTP instead of TTP along with uracil‐DNA glycosylase prior to guard against accidental amplicon contamination. An internal amplification control (gBlocks Gene Fragment from Integrated DNA Technologies) was prepared in 1 µg/ml of calf thymus DNA in TRIS‐EDTA, pH 8 (Fisher catalog # BP2473‐1) and added to all samples to monitor potential PCR inhibition. gBlocks covering all target amplicon sequences were used as positive PCR control samples.

PCR was performed with Fluidigm's BioMark HD System using 96.96 Dynamic Array™ IFC Arrays, a microfluidic system capable of analyzing 96 samples with 96 separate PCR assays. Each PCR contained 3 nl of extracted DNA plus 610 nmol/L each PCR primer, 340 nmol/L fluorescent reporter probe, and 0.91X ThermoFisher TaqPath qPCR MasterMix, CG (P/N A16245). PCR was performed with the following cycling program: 2 min at 50°C, 10 min at 95°C and 40 cycles of 15 s at 95°C, and 1 min at 60°C.

### 2.5 Statistical analysis

Phenotypic and genotypic AR were summarized using frequencies and percentages. The percent of organisms with AR to various antimicrobial agents in the Chesapeake Bay were compared to those in the Upper Tributary using the Fisher's exact test. Statistical analysis was performed using SAS version 9.4 (SAS Institute, Inc., Cary, NC). All tests were two‐sided and *p* < 0.05 was considered statistically significant.

## 3 RESULTS

A total of 92 different gram‐negative enteric bacteria were isolated from all sites; 51 bacterial isolates (55%) were recovered from the various collection sites around the Chesapeake Bay (CB), and 41 bacterial isolates (45%) were recovered from the adjacent upper tributaries (UT) (Table 1). Overall, the highest numbers of organisms were recovered from the Potomac River and Sandy Point State Park and represented 15.2% (14/92) and 13.0% (12/92) of the total, respectively. In comparison, the lowest number of recovered isolates was seen in samples taken from the Monocacy River (4/92; 4.3%). Organisms most frequently isolated across all sampling sites included *K. pneumoniae* (*n* = 17; 18%); *Enterobacter cloacae* (*n* = 16; 17%); *Enterobacter aerogenes* (*n* = 15; 16%); *Serratia marcescens* (*n* = 15; 16%); *Escherichia coli* (*n* = 13; 14%). *Klebsiella pneumoniae* and *Enterobacter cloacae* were more frequently isolated from samples of the CB (76% and 81%, respectively) than the UT (24% and 19%, respectively), whereas *S. marcescens* was more frequently isolated in samples from the UT (93%) than the CB (7%). These differences were statistically significant (*p* < 0.01). No statistically significant difference in frequency of isolation from CB and UT sampling sites was observed for *Enterobacter aerogenes* and for *Escherichia coli*; however, *Escherichia coli* was slightly more often recovered from the UT (62%) than from the CB (38%), yet the difference was not statistically significant (*p* = 0.23). For sampling sites from the CB, *Enterobacter cloacae* was more often recovered in water samples collected in close proximity to the shore (70%), whereas *Escherichia coli* was predominantly recovered in samples collected distally to the shore (80%).

**Table 1:** Gram‐negative bacterial isolates recovered from the various sampling sites on the Chesapeake Bay and the adjacent watershed

Bacterial Organism [*N*; %] [total *N* = 92]	Chesapeake Bay sampling sites (*N* = 51; 55.4%)										Upper Tributary sampling sites (*N* = 41; 44.6%)				
	North Point State Park		Baltimore Inner Harbor		Eastern Neck Wildlife Preserve		Sandy Point State Park		Gunpowder State Park		Monocacy River	Catoctin Creek	Potomac River	Shenandoah River	Point of Rocks
	Proximal	Distal	Proximal	Distal	Proximal	Distal	Proximal	Distal	Proximal	Distal					
*Klebsiella pneumoniae *[17; 18.5%]	0	0	3	3	1	0	1	1	2	2	1	1	2	0	0
*Enterobacter cloacae *[16; 17.4%]	3	0	3	0	0	1	1	1	2	2	0	0	3	0	0
*Enterobacter aerogenes *[15; 16.3%]	0	1	0	0	2	0	1	4	0	0	1	1	0	5	0
*Serratia marcescens *[15; 16.3%]	0	0	0	0	0	0	0	1	0	0	0	3	5	1	5
*E. coli *[13; 14%]	0	2	0	1	1	0	0	1	0	0	0	3	3	2	0
*Citrobacter freundii *[7; 7.6%]	4	0	0	0	0	0	0	0	0	0	2	0	0	0	1
*Morganella morganii *[2; 2.2%]	0	0	0	1	0	0	0	0	0	0	0	0	1	0	0
*Enterobacter sakazakii *[1; 1%]	0	0	0	0	0	0	0	0	1	0	0	0	0	0	0
*Pantoeae* spp. [1; 1%]	0	1	0	0	0	0	0	0	0	0	0	0	0	0	0
*Raoultella *spp. [1; 1%]	0	0	0	0	1	0	0	0	0	0	0	0	0	0	0
*Citrobacter braakii *[1; 1%]	0	0	0	0	0	1	0	0	0	0	0	0	0	0	0
*Citrobacter youngii *[1; 1%]	0	0	0	0	0	0	0	0	0	0	0	0	0	0	1
*Aeromonas hydrophila *[1; 1%]	0	0	0	0	0	0	1	0	0	0	0	0	0	0	0
*Plesiomonas shigelloides *[1; 1%]	0	0	0	0	0	0	0	0	0	1	0	0	0	0	0
Total number of isolates (%)	7 (7.6%)	4 (4.4%)	6 (6.5%)	5 (5.4%)	5 (5.4%)	2 (2.2%)	4 (4.4%)	8 (8.7%)	5 (5.4%)	5 (5.4%)	4 (4.4%)	8 (8.7%)	14 (15.2%)	8 (8.7%)	7 (7.6%)

Results for the phenotypic detection of AR are shown in Tables 2 and 3. Seventy‐eight percent of all isolates (70/90) tested against ampicillin were found to have tested either intermediately susceptible [I] or resistant [R]; of those isolates that were “resistant” to ampicillin, 57/70 (81%) were found to be resistant, whereas 13/70 (19%) were found to be intermediate susceptible. No statistically significant difference was observed between bacteria isolated from the CB versus the UT. When tested against chloramphenicol, 28/92 (30.4%) isolates were found to be resistant (I + R); eight isolates were resistant and 20 isolates were intermediate‐susceptible. While the total number of isolates that tested either intermediate or resistant to chloramphenicol was the same for CB ant UT, a slightly higher rate of resistance to chloramphenicol was found among organisms isolated from the UT (14/41; 34.1%) compared to the organisms isolated from the CB (14/51; 27.5%). A total of 40.2% of all isolates (*n* = 37) were found to be resistant (I + R) to tetracycline, with 48.8% of organisms recovered from UT sampling sites being resistant to tetracycline, and only 34.7% of isolates from CB sampling sites. Of these 37 isolates with tetracycline resistance, 10 were found to be intermediate‐susceptible, and 27 were resistant. With respect to the carbapenems tested in this study, only imipenem resistance (I + R) was detected at a slightly higher rate when compared to all other carbapenems, with eight isolates being intermediate‐susceptible and seven isolates being resistant. Overall resistance to imipenem was found in 16.7% of all isolates, with imipenem resistance in organisms recovered from the CB being significantly higher (24.5%) when compared to isolates recovered from the UT (7.3%); this difference was statistically significant (*p* = 0.045). A total of four isolates were found to have some resistance (I + R) to ertapenem (*K. pneumoniae* [R]; *Enterobacter cloacae* [I]; *Morganella morganii* [I]; *Pantoea* [I]). One isolate (*Morganella morganii*), recovered from the Potomac River, tested resistant to doripenem; it was also resistant to imipenem and intermediate to ertapenem, but susceptible to meropenem. However, the meropenem MIC for this isolate was within one‐doubling dilution from the breakpoint to being “intermediate‐susceptible.” While generally no resistance to fluoroquinolones (specifically ciprofloxacin) was detected among all isolates in this study, one *Pantoea* isolate recovered from the CB tested intermediate against ciprofloxacin. Sporadically, some bacterial isolates tested resistant to ceftriaxone, ceftaroline, and cefepime. Three isolates recovered from sites at the CB were resistant [R] to cefepime, and no cefepime resistance was seen in isolates from the UT. Two isolates from the CB and two isolated from the UT were found to be resistant [R] to ceftriaxone; no isolate tested intermediate‐susceptible. Two isolates from the CB were resistant [R] to ceftaroline and one isolate was found to be intermediate‐susceptible; of the five isolated recovered from the UT sites, three were found to be intermediate‐susceptible to ceftaroline, and two were resistant [R]. However, the overall frequency and rates of these resistances was negligible, considering all isolates recovered from the various sites in this study. Similarly, only one isolate recovered from CB samples tested resistant to piperacillin/tazobactam, and two other isolates tested intermediate‐susceptible. Finally, no resistance to aminoglycosides tested in our study (gentamicin and amikacin) was detected in the bacterial organisms isolated from the CB and UT.

**Table 2:** Antimicrobial resistance by phenotypic AST methods detected in gram‐negative bacterial organisms, listed by antimicrobial agent and geographic site of isolation

Antimicrobial agent [MIC (µg/mL) interpretation]^a^	Modal MIC (µg/mL) [Range]	
	Chesapeake bay sampling sites	Upper tributary sampling sites
Ampicillin ≤ 8: S; 16: I; ≥32: R	16 [4–≥256]	32 [8–≥256]
Piperacillin/Tazobactam ≤ 16/4: S; 32/4 – 64/4: I; ≥128/4: R	4 [0.5–64]	2 [1–8]
Ceftriaxone ≤ 1: S; 2: I; ≥2: R	0.25 [0.047–1]	0.125 [0.047–0.25]
Ceftaroline ≤ 0.5: S; 1: I; ≥4: R	0.25 [0.006–0.5]	0.5 [0.064–0.25]
Cefepime ≤ 2: S; 4–8: SDD; ≥16: R	0.064 [0.047–1]	0.064 [0.047–1]
Ertapenem ≤ 0.5: S; 1: I; ≥2: R	0.032 [0.012–1]	0.032 [0.004–1]
Imipenem ≤ 1: S; 2: I; ≥4: R	0.5 [0.125–16]	0.5 [0.125–8]
Meropenem ≤ 1: S; 2: I; ≥4: R	0.064 [0.012–8]	0.064 [0.012–0.064]
Doripenem ≤ 1: S; 2: I; ≥4: R	0.064 [0.023–1]	0.064 [0.012–4]
Gentamicin ≤ 4: S; 8: I; ≥16: R	1 [0.25–8]	0.5 [0.125–4]
Amikacin ≤ 16: S; 32: I; ≥64: R	4 [2–16]	4 [1–4]
Ciprofloxacin ≤ 1: S; 2: I; ≥4: R	0.032 [0.016–0.25]	0.032 [0.016–0.25]
Tetracycline ≤ 4: S; 8: I; ≥16: R	4 [1 ‐ ≥256]	4 [1 ‐ ≥256]
Trimethoprim/Sulfamethoxazole ≤ 2/38: S; ≥4/76: R	0.125 [0.064–2]	0.25 [0.064–0.5]
Chloramphenicol ≤ 8: S; 16: I; ≥32: R	8 [0.5–256]	8 [2–128]

a MIC interpretive criteria, based on CLSI M100‐S25; Clinical and Laboratory Standards Institute (CLSI). (2016); S: susceptible; SDD: susceptible, dose‐dependent; I: intermediate susceptible; R: resistant.

**Table 3:** Phenotypic antimicrobial resistance (I + R) of bacteria, overall and by location^a^

Antimicrobial Agent tested against GNR isolated (*N* = 92)	Antimicrobial Resistance (I + R) by Location *N* (%)			
	All Locations *N* = 92 (%)	Chesapeake Bay *N* = 51 (55.5%)	Upper Tributary *N* = 41 (44.5%)	*p* value
Ampicillin (*n* = 90)	70 (78)	38 (78)	32 (78)	0.99
Ceftaroline	8 (9)	3 (6)	5 (12)	0.46
Ceftriaxone	4(4)	2 (4)	2 (5)	0.99
Cefepime	3 (3)	3 (6)	0 (0)	0.25
Imipenem	15 (17)	12 (24)	3 (7)	0.045
Doripenem	1 (1)	0 (0)	1 (2)	0.46
Ertapenem (*n* = 90)	4 (4)	3 (6)	1 (2)	0.62
Meropenem	2 (2)	2 (4)	0 (0)	0.16
Pip/Tazo (*n* = 88)	3 (3)	3 (6)	0 (0)	0.50
Chloramphenicol	28 (31)	14 (29)	14 (34)	0.65
Amikacin	0 (0)	0 (0)	0 (0)	—
Gentamicin	1 (1)	1 (2)	0 (0)	0.99
Ciprofloxacin	1 (1)	1 (2)	0 (0)	0.99
Tetracycline	37 (41)	17 (35)	20 (49)	0.20

Note

*p *value is from Fisher's exact test; *p* < 0.05 is considered significant.

Pip/Tazo: Piperacillin/Tazobactam.

GNR: Gram‐negative rod (bacteria); one *Aeromonas* isolate and one *Plesiomonas* isolate were not tested against ampicillin, ertapenem as per CLSI guidelines.

a Antimicrobial resistance (I + R) based on MIC interpretive criteria of CLSI M‐100 S25 (2016).

Detection of select genotypic resistance and distribution of resistance genes (ACT‐1/MIR‐1; CMY‐2/CFE‐1; CMY‐47; CMY‐70/CFE‐1; MOX‐1/CMY‐1; SHV‐G156; SHV‐G238; OXA‐50; DHA‐1) among the various bacterial organisms and the sites of organism recovery are shown in Tables 4 and 5. Overall, resistance genes were detected in 71% (35/49) of organisms isolated from CB sample sites, while only 27% (11/41) of all organisms isolated from UT sample sites (Table 5) had resistance genes detected. This difference was statistically significant (*p* < 0.001). The CMY‐2 and CMY‐70 genes, both of which belong to large families of plasmid‐mediated *Amp‐C* β‐lactamases (cephamycinases), were most frequently identified in isolates of *Escherichia coli* (7/8) and *Citrobacter freundii* (3/3) recovered from UT sites in the adjacent watershed and rivers. One isolate of *Morganella morganii* from the Potomac River was carrying the DHA‐1 gene, which encodes for a plasmid‐mediated *AmpC *β‐lactamase (cephalosporinase). One isolate of *K. pneumoniae* from the Monocacy River was positive for both the SHV‐G156 and SHV‐G238 genes. The SHV‐G156 and SHV‐G238 genes encode for a large family of chromosomal‐ as well as plasmid‐encoded β‐lactamases. These enzymes hydrolyze narrow‐spectrum cephalosporins, penicillins, and aztreonam, and can be blocked by the β‐lactamase inhibitor clavulanic acid. From the various CB sites, several plasmid‐mediated as well as chromosomally encoded *AmpC* β‐lactamase genes (ACT‐1/MIR‐1, CMY‐2, CMY‐47, CMY‐70) were detected in various organisms (*Escherichia coli*, *C. freundii*, and *Enterobacter* species); in addition, the SHV‐G156 and SHV‐G238 genes were detected in 92% (12/13) and 54% (7/13) of *K. pneumoniae *isolates, respectively. In addition, the SHV‐G156 gene was also detected in one isolate of *Enterobacter aerogenes* and *Raoultella terrigena*. The predominance of detecting the SHV‐G156 gene in isolates from the CB sites compared to the UT sites was statistically significant (*p* = 0.001). In addition, the difference in detection of the ACT‐1 gene isolates from the CB compared to the UT sampling sites was also statistically significant (*p* = 0.007; see Table 5).

**Table 4:** Resistance genes detected by the Acuitas test in gram‐negative bacterial organisms, listed by bacterial organisms and geographic site of isolation

Bacterial Organism (# isolated)	Chesapeake Bay sampling sites					Upper Tributary sampling sites				
	North Point State Park	Baltimore Inner Harbor	Eastern Neck Wildlife Preserve	Sandy Point State Park	Gunpowder State Park	Monocacy River	Catoctin Creek	Potomac River	Shenandoah River	Point of Rocks
*Klebsiella pneumoniae*	“x”	SHV‐G156 SHV‐G238	SHV‐G156	SHV‐G156 SHV‐G238	SHV‐G156	SHV‐G156 SHV‐G238	“x”	“x”	“x”	“x”
*Enterobacter cloacae*	ACT‐1/MIR‐1 CMY‐2/CFE‐1 CMY‐70	ACT‐1/MIR‐1	“x”	ACT‐1/MIR‐1	ACT‐1/MIR‐1 CMY‐2/CFE‐1 CMY‐70 CMY‐47	“x”	“x”	“x”	“x”	“x”
*Enterobacter aerogenes*	“x”	“x”	“x”	SHV‐G156	“x”	“x”	“x”	“x”	“x”	“x”
*Enterobacter sakazakii*	“x”	“x”	“x”	“x”	ACT‐1/MIR‐1	“x”	“x”	“x”	“x”	“x”
*E. coli*	CMY‐2/CFE‐1 CMY‐70	CMY‐2/CFE‐1 CMY‐70	CMY‐2/CFE‐1 CMY‐70	“x”	“x”	“x”	CMY‐2/CFE‐1 CMY‐70	CMY‐2/CFE‐1 CMY‐70	CMY‐2/CFE‐1	“x”
*Citrobacter freundii*	CMY‐2/CFE‐1 CMY‐47	“x”	“x”	“x”	“x”	CMY‐2/CFE‐1 CMY‐70 CMY‐47	“x”	“x”	“x”	CMY‐2/CFE‐1 CMY‐70 CMY‐47
*Citrobacter braakii*	“x”	“x”	CMY‐2/CFE‐1 CMY‐70 CMY‐47	“x”	“x”	“x”	“x”	“x”	“x”	“x”
*Morganella morganii*	“x”	CMY‐2/CFE‐1 CMY‐70	“x”	“x”	“x”	“x”	“x”	DHA‐1	“x”	“x”
*Raoultella *spp.	“x”	“x”	SHV‐G156	“x”	“x”	“x”	“x”	“x”	“x”	“x”
*Aeromonas hydrophila*	“x”	“x”	“x”	MOX‐1	“x”	“x”	“x”	“x”	“x”	“x”

Note

ACT‐1/MIR‐1, CMY‐2/CFE‐1, CMY‐70/CFE‐1, DHA‐1, MOX‐1: plasmid‐mediated Amp‐C beta‐lactamases; CMY‐47: chromosomal‐encoded Amp‐C beta‐lactamase; SHV‐G156 and SHV‐G238: chromosomally‐ and plasmid‐encoded β‐lactamases; “x”: no resistance gene detected by Acuitas AMR panel.

**Table 5:** Genotypic detection of antimicrobial resistance of bacteria, overall and by location

Resistance Gene	All Locations *n* = 90 (%)	Chesapeake *n* = 49 (%)	Upper Tributary *n* = 41 (%)	*p* value
Any	46 (51)	35 (71)	11 (27)	<0.001
ACT‐1	8 (9)	8 (16)	0 (0)	0.007
CMY‐2	22 (24)	12 (24)	10 (24)	0.99
CMY‐47	9 (10)	6 (12)	3 (7)	0.50
CMY‐70	16 (18)	8 (16)	8 (20)	0.78
DHA‐1	1 (1)	0 (0)	1 (2)	0.46
MOX‐1	0 (0)	0 (0)	0 (0)	—
OXA‐50	0 (0)	0 (0)	0 (0)	—
SHV‐G156	15 (17)	14 (29)	1 (2)	0.001
SHV‐G238	8 (9)	7 (14)	1 (2)	0.07

Note

*p* value is from Fisher's exact test; *p* < 0.05 is considered significant.

Resistance genes: ACT‐1/MIR‐1, CMY‐2/CFE‐1, CMY‐70/CFE‐1, DHA‐1, MOX‐1: plasmid‐mediated Amp‐C β‐lactamases; CMY‐47: chromosomal‐encoded Amp‐C β‐lactamase; SHV‐G156 and SHV‐G238: chromosomally‐ and plasmid‐encoded β‐lactamases.

## 4 DISCUSSION

This study investigated the occurrence of AR in gram‐negative bacteria isolated from various sample sites at the Maryland CB and its adjacent UT. We identified resistance against several antimicrobial agents in a variety of gram‐negative Enterobacteriaceae isolated from various surface water samples from a variety of sampling sites surrounding the CB and UT. Generally, a larger number of bacterial organisms were recovered from the sampling sites in the CB (including the Baltimore Inner Harbor) when compared to the UT sampling sites. Interestingly, phenotypic AST revealed generally no statistically significant difference in distribution of antimicrobial resistant organisms between the CB and UT sampling sites, albeit, a statistically significant difference was observed for imipenem resistance. Imipenem resistance was more often observed in organisms isolated from the CB samples. However, a statistically significant difference was more frequently observed in the detection of genotypic resistance from organisms isolated from the CB samples compared to the UT samples; this difference was most pronounced for the detection of plasmid mediated *AmpC* β‐lactamases as well as the plasmid mediated SHV‐type extended‐spectrum β‐lactamases. The results from our study demonstrate that Enterobacteriaceae occurring in surface waters are important reservoirs for AR for a number of antibiotic classes, including β‐lactam antimicrobial agents, carbapenems, tetracyclines, and chloramphenicol. Several of the isolates showed high levels of resistance to select antimicrobial agents, specifically some of the β‐lactam antimicrobial agents. The antimicrobial agents that were tested in this study represent those that may be typically used to treat a variety of clinical infections. Some of these infections (e.g., wound infections) could be acquired through recreational activities when wounds become contaminated with water, soil, or other environmental sources, which may in turn contain bacteria that are resistant to the various antimicrobial agents that one would use for treatment. Furthermore, bacteria that are harboring AR and that are present in surface waters could be the source for transferring such resistance genes to yet other, still susceptible bacteria that are present in the water. Lastly, surface waters may not be just be important because of human recreational activities, but surface waters and wastewaters are also affecting agriculture and animal husbandry; in these situations, antimicrobial‐resistant bacteria may be further spread among animals for food production. The threat to human and global health is significant: humans may become infected by AR bacteria from livestock, or by consumption of contaminated food and water; humans may also become colonized with such AR bacteria; and finally, AR may be spread through the above referenced means on a more global scale (Wattkins & Bonomo, 2016).

Recreational water and wastewater have specifically been identified as a potential source of resistant bacteria in the environment that could affect the gut and other microbiota of wildlife (Aminov, 2009; Aminov & Mackie, 2007; Campagnolo et al., 2002; Martinez, 2009); however, these bacteria potentially pose a risk to human health as well, specifically in areas prone to recreational activities (e.g. fishing and other water related sports activities). We had previously reported a case of an unusual multidrug‐resistant *Mycobacterium marinum* isolated from a patient with a soft tissue infection following a fish‐hook injury after fishing activities on the Chesapeake Bay (Parrish et al., 2011). With regard to mycobacteria, *Mycobacterium marinum* is not the only species of mycobacteria to have been recovered from the CB. A single isolate of *Mycobacterium cosmeticum* with unusual AR was isolated from the Sandy Point State Park sampling site (Atukorale, Boire, Dionne, Riedel, & Parrish, 2017). This isolate demonstrated resistance to a number of antibiotics including doxycycline, tigecycline, clarithromycin, trimethoprim/sulfamethoxazole, imipenem, cefoxitin, ethionamide, and streptomycin versus isolates of this species described in previously published reports which were pan‐susceptible. One other study investigated the presence of AR in *Vibrio* species isolated from water samples from the CB (Shaw et al., 2014). The investigators found that a significant number of *Vibrio* isolates were resistant to chloramphenicol despite the fact that most isolates of *V. vulnificus* and *V. parahaemolyticus* recovered in their study remained susceptible to antimicrobial agents commonly used to treat infections due to *Vibrio* species (Shaw et al., 2014). Furthermore, several isolates in this study tested had a low‐level, intermediate resistance to third‐ and fourth‐generation cephalosporins. In a similar study the investigators found the presence of unusual resistance patterns in *Aeromonas hydrophila* isolates from various aquatic environments, including the Chesapeake Bay (McNicol et al., 1980). While in this study *A. hydrophila* isolates from various aquatic environments outside the United States readily demonstrated chloramphenicol resistance, the *A. hydrophila* isolates from the CB remained susceptible to chloramphenicol; however, all of the CB isolates were resistant to ampicillin, and also demonstrated a high level of resistance to tetracycline (McNicol et al., 1980). Furthermore, the authors commented on the fact that water samples from various sites of the CB had a high level of pollution with enteric gram‐negative bacteria. Interestingly, these findings are consistent with the findings in one of the earlier studies investigating the water quality and AR in the CB (Morgan et al., 1976). In recent years, investigations of water quality and specifically the detection of AR in enteric bacteria as well as other bacteria commonly isolated from aquatic environments have been recognized as important components of monitoring AR evolution in the greater context of the One Health Initiative (Allen et al., 2011; Edge & Hill, 2005; Hamelin et al., 2006; Stange, Sidhu, Tiehm, & Toze, 2016; Wright, 2010; One Health Initiative, 2018). One Health has been defined as “the collaborative effort of multiple disciplines working locally, nationally, and globally to attain optimal health for people, animals, and the environment” (One Health Initiative, 2018). Here, we specifically referenced studies investigating the detection of bacterial organism burden and AR in various aquatic environments as a comparison to our study design. In the studies referenced here, the investigators detected predominantly resistance to the following classes of antimicrobial agents: ampicillin/amoxicillin, tetracyclines, trimethoprim/sulfamethoxazole; it is of note that resistance to aminoglycosides and chloramphenicol was also detected in some bacterial organisms (e.g., *Vibrio*) but not in others (e.g., *Aeromonas*). In comparison to all these referenced studies, the findings in our study of the CB and UT isolates have similarities to the findings in some but not all of the other studies, specifically with respect to identifying resistance to ampicillin/amoxicillin, tetracycline, and chloramphenicol. It is also of interest to note that the majority of the isolates in our current study demonstrated resistance to chloramphenicol, whereas bacterial isolates from earlier studies (McNicol et al., 1980; Morgan et al., 1976) of CB water samples did not detect such resistance with the exception of Shaw et al. whose study described chloramphenicol resistance in *Vibrio *species (Shaw et al.., 2014). While such differences in results from these various studies are readily apparent, it is important to consider that most studies, including our own study, were only conducted during a limited time period and/or season. None of these studies present data for longitudinal, long‐term, ongoing surveillance over the course of an entire year or even years. Furthermore, most studies focused on specific groups of bacteria, for example, *Vibrio *species (Shaw et al., 2014), *Aeromonas* species (McNicol et al., 1980)*, *Enterobacteriaceae* (*Morgan et al., 1976). It is likely that the diversity of bacterial organisms present in various aquatic environments will undergo seasonal changes and is further influenced by a variety of other factors (e.g. human activities, environmental factors, weather events, etc.). Additional studies that also include longitudinal surveillance of AR will be necessary to establish a better and more in‐depth understanding of the AR in bacterial organisms in various aquatic environments and their subsequent potential impact on human health. Considering the One Health concepts with respect to emerging AR in various clinical and nonclinical settings and its importance to human health, we believe that our data underscore the importance of efforts to monitor aquatic and other environments with respect to the presence of enteric and other bacteria with AR, as such bacteria are likely to contribute to the growing burden of AR globally. Humans continue to be exposed to aquatic and other environments through various activities, including recreational activities of all kinds, and one should recognize the potential of accidental injuries with subsequent exposure and possible infection with pathogenic bacteria with higher levels of AR. The CB is North America's largest and most biologically diverse estuary; however, since the 1970s its water quality has declined significantly (Ruhl & Rybicki, 2010; Savage & Ribaudo, 2013; Wainger, 2012). This decline of water quality has been largely attributed to the human population increase as well as aggressive agricultural production and animal husbandry practices (Bernhardt & Pelton, 2017; Land, 2012; Randall, 2003). Since the 1980s, the CB and its adjacent watershed has been the focus of numerous State and Federal initiatives, mainly focused on the reduction of nutrient pollution from agriculture and other sources of human activities. Success of such measures has been reported to a limited extent (Ruhl & Rybicki, 2010; Savage & Ribaudo, 2013; Wainger, 2012). The results from our study demonstrate that in various locations around the CB, a significant amount of enteric bacteria are present in the surface water. Such bacteria are likely to originate from human and animal biowaste; in addition, we identified the presence of AR against various antimicrobial agents in these bacteria. Some of these antimicrobial agents are commonly used in animal husbandry as growth promoters and to prevent and/or treat infections of farm animals.

We recognize that our study has several limitations; samples were collected only at a single point in time, and no consecutive sampling was performed. Furthermore, our study focused on gram‐negative bacteria, specifically Enterobacteriaceae, which were characterized for genetic determinants of resistance which indicated differences between most strains suggesting they were not clonal isolates. A more detailed genomic characterization of each isolate was beyond the scope of this study and was not performed, thus the extent of clonality was not determined. However, these limitations may suggest potential avenues for future research to augment the understanding of emerging AR in bacterial isolates from various environmental sources in relation to human activities.

In summary, the results from this study contribute to a better understanding of AR and the mobility of resistance genes in organisms isolated from aquatic environments, specifically those in close proximity to areas of human recreational and other activities, as well as animal husbandry activities. While measures for management and control of water quality have been partly implemented, our data suggest that such initiatives could be augmented and broader surveillance of water quality should also include the assessment of bacterial enteric burden together with surveillance of antimicrobial resistance in such bacterial isolates. Longitudinal, sustained surveillance studies may be necessary to further enhance our understanding of the complex issues related to the emergence of antimicrobial resistance at the interface been the environment and human activities.

## CONFLICT OF INTERESTS

Dr. Stefan Riedel serves as a member of the Clinical Advisory Board and as a paid consultant to OpGen, Inc., which provided the Acuitas Resistome Test for the analysis of the resistance genes identified in the bacterial organisms in this study. The mention of trade names or commercial products in this publication is solely for the purpose of providing specific information and does not imply recommendation and/or endorsement of these products by the authors, singly or collectively.

## AUTHOR CONTRIBUTIONS

S.R., N.B., and N.M.P. conceived and designed the experiments, and contributed to the writing of the manuscript; N.B., A.V., J.K., V.V., V.A., V.A.A.R., S.R., and N.M.P. conducted the experiments, including the water sampling, and all or parts of the laboratory‐based experiments; K.A.C. conducted the statistical analyses and contributed to the writing of the manuscript.

## ETHICS STATEMENT

The study protocol was reviewed and approved by the Johns Hopkins University, School of Medicine, Institutional Review Board (IRB) and considered non‐human subject research. Therefore no further IRB review was required.

## Data Availability

The data that support the findings of this study are not publicly available. However, data are available from the authors upon reasonable request and with permission of Drs. Nicole Parrish and Stefan Riedel, who are the co‐principal investigators of this study.
